# Exploring the Association between Gut and Urine Microbiota and Prostatic Disease including Benign Prostatic Hyperplasia and Prostate Cancer Using 16S rRNA Sequencing

**DOI:** 10.3390/biomedicines10112676

**Published:** 2022-10-23

**Authors:** Kai-Yen Tsai, Deng-Chyang Wu, Wen-Jeng Wu, Jiunn-Wei Wang, Yung-Shun Juan, Ching-Chia Li, Chung-Jung Liu, Hsiang-Ying Lee

**Affiliations:** 1 Department of Post-Baccalaureate Medicine, College of Medicine, Kaohsiung Medical University, Kaohsiung 80708, Taiwan; 2 Division of Gastroenterology, Department of Internal Medicine, Kaohsiung Medical University Hospital, Kaohsiung Medical University, Kaohsiung 80708, Taiwan; 3 Regenerative Medicine and Cell Therapy Research Center, Kaohsiung Medical University, Kaohsiung 80708, Taiwan; 4 Department of Medicine, Faculty of Medicine, College of Medicine, Kaohsiung Medical University, Kaohsiung 80708, Taiwan; 5 Department of Urology, Kaohsiung Medical University Hospital, Kaohsiung 80708, Taiwan; 6 Department of Urology, School of Medicine, College of Medicine, Kaohsiung Medical University, Kaohsiung 80708, Taiwan; 7 Graduate Institute of Clinical Medicine, College of Medicine, Kaohsiung Medical University, Kaohsiung 80708, Taiwan; 8 Urology Department, Kaohsiung Municipal Ta-Tung Hospital, Kaohsiung 80145, Taiwan

**Keywords:** microbiota, prostatic hyperplasia, prostatic neoplasms, sequence analysis, RNA

## Abstract

Numerous microorganisms residing in the gastrointestinal and genitourinary tracts affect host health. We investigated stool and voided urine samples collected from patients with benign prostatic hyperplasia (BPH) or prostate cancer (PC) and a control group to explore the potential relationship between human microbiota and prostatic disease, and aimed to identify correlations and pathogenic taxonomic units. We studied microbial composition using 16S rRNA sequencing to identify operational taxonomic units (OTUs). Extracted genome was amplified and filtered sequences were used to classify OTUs based on their specific taxonomy. No statistically significant differences were observed in stool samples among the groups. However, urine samples indicated different microbiota compositions in different patient populations. The top five microbial genera that showed significant differences between the BPH and control groups were *Alcaligenes*, *Pseudomonas*, *Lactobacillus*, *Akkermansia*, and *Cetobacterium*. *Faecalibacterium*, *Staphylococcus*, *Ruminococcaceae_UCG_002*, *Neisseria*, and *Agathobacter* were the genera with the largest proportion differences when comparing the PC and control groups. We discovered that the urine microbiota composition of the BPH and PC groups was distinct from that of the control group. Due to the impact of microbiota on prostatic disease, it is necessary to identify specific microbes for further research.

## 1. Introduction

The microbiome plays a complex role in human cancer. Cancer progression is sometimes linked to microorganisms located within, adjacent to, or distant from tumors [1]. Disturbances in the composition of certain microorganisms have been linked to urologic disorders [2]. In addition to the genitourinary tract, numerous microorganisms residing in the gastrointestinal tract may also affect host health. In the human body, the gut shows a significantly diverse microbial community and contains at least 1000 species of microbiotas [3]. Aberrant intestinal microbial behavior is related to pathological changes in the host intestine and colorectal cancer [4].

Benign prostatic hyperplasia (BPH) induces lower urinary tract symptoms (LUTS), which often occur in middle-aged and elderly people. Approximately 80% of men aged > 70 years experience BPH and LUTS [5]. BPH is one of the most common urological diseases worldwide. In 2015, 12.9 million men consulted physicians for BPH in the United States [6]. Prostate cancer (PC) accounts for 26% of newly diagnosed cancers and is the second leading cause of death among cancers in the United States [7]. The prevalence rate of PC is 16% in American males and accounts for 3% of all deaths [8]. Merely within one year after the diagnosis of PC, Medicare payments for PC care expend approximately USD 20,000–30,000 per patient [9]. Both BPH and PC deserve urologists’ attention in developing more efficient disease prevention strategies, diagnostic methods, and treatment.

Mature sequencing techniques have empowered scientists and urologists to shed light on the possible correlation between the microbiota in the genitourinary tract and diseases. Yu et al. investigated urine, seminal fluid, and prostatic secretions collected from patients with BPH or PC. Diverse microbial populations were reported in PC and BPH samples [10]. A similar result was obtained in 2017. Shrestha et al. collected urine samples from male patients before they underwent PC biopsy. The research suggested that pro-inflammatory bacteria and uropathogens are present in the urinary tract of patients with PC [11]. Our study aimed to explore the potential relationship between the human microbiota and prostatic diseases, including BPH and PC. Before the study was published, there were very few studies on BPH, PC, and normal cohort microbiota within a single study. In addition, an adequate sample size makes the study statistically significant. This article points out several genera that thrive in the body of prostatic patients. This could help researchers to focus on specific genera in future studies.

## 2. Materials and Methods

### 2.1. Patient Population and Specimen Collection

Urine and stool specimens were collected from three groups: patients with BPH, patients with PC, and a control group. None of these participants had taken antibiotics within the past two months or had a recent history of urethral catheter insertion. There were 77, 62, and 46 urine specimens obtained from patients with BPH, patients with PC, and the control group, respectively. A total of 75, 59, and 36 stool specimens were obtained from patients with BPH, patients with PC, and the control groups, respectively. Enrolled patients with BPH were under medical treatment for LUTS. The enrolled PC patients were pathologically diagnosed, and the stage ranged from Ia to IIIb PC. The control group were males without BPH (with LUTS) or PC. Patients with comorbidities that may be related to LUTS, including neurological disorders and infections, were also excluded. The stool was collected from patients in clean and dry containers. The collected stool specimens were mixed with the preservation solution in a sterile kit at 25°C. Urine was preserved in a freezer at −80°C.

### 2.2. Demographics Recording of Patients

In addition to the participants’ demographics, we used questionnaires including the International Prostate Symptom Score (IPSS) and Quality of Life Scale (QOLS) to evaluate the severity of LUTs. We collected clinical hematology data on fasting blood glucose (Ac sugar), glycated hemoglobin (HbAlc), cholesterol, triglyceride (TG), low-density lipoprotein (LDL), high-density lipoprotein (HDL), glutamic-oxaloacetic transaminase (GOT), glutamic-pyruvic transaminase (GPT), blood urea nitrogen (BUN), creatinine (Cr), prostate-specific antigen (PSA), free PSA, testosterone, and insulin. Ultrasonography was also used to measure prostate gland size.

### 2.3. Specimen Sequencing and Molecular Methods

Using the CTAB/SDS method, genome was extracted from urine and stool samples and diluted to 1 ng/μL with sterile water. The DNA concentration and purity were monitored on a 1% agarose gel. The conserved and hypervariable regions of the bacterial 16S rRNA were our target sequences. A polymerase chain reaction (PCR) of the conserved region was performed to amplify the sequence. The 16S rRNA genes of distinct regions (16SV4/16SV3/16SV3-V4/16SV4-V5) were amplified using specific primers (e.g., 16S V4: 515F–806R). All PCR reactions were performed using the Phusion High-Fidelity PCR Master Mix. Electrophoresis was performed on 2% agarose gel for detection. PCR products were mixed in equidensity ratios, and then the PCR products were mixed using the Qiagen Gel Extraction Kit.

The hypervariable regions were included in the amplified sequences. FLASH (v1.2.11) was used to assemble 300 bp paired-end raw reads to obtain a raw tag. We used the Qiime 1.9.1 pipeline for quality control to discard tags with three consecutive bases’ Q less than 19 or less than 75% of the original length. We also filtered the chimera sequences using the UCHIME algorithm to obtain an effective tag. Effective tags were then clustered as operational taxonomic units (OTUs) using the UPARSE algorithm of the USEARCH software (v7.0.1090), and a 97% identity threshold was set for categorizing OTUs. We used the RDP Classifier (v2.2) to classify the OTUs based on their specific taxonomy. The database accessed by the RDP Classifier was the Silva Database (v.132).

### 2.4. Statistical Analysis

ANOSIM was used to determine whether there was a significant difference in the microbial community among the three groups. R vegan package (version 3.3.1) was used to perform the ANOSIM function. R was also used to draw the rarefaction curve to show a sufficient number of samples. Partial least squares discriminant analysis (PLS-DA) was used to study beta diversity. PLS-DA was conducted using the mixOmics and ggplot2 packages in the R software (ellipse level = 0.3). The R ggtern package was used to generate ternary plots. The top 30 abundant OTUs were selected to draw a Spearman correlation coefficient plot at the genus level. We used the R corrplot package to conduct the Spearman correlation coefficient analysis. We utilized statistical analysis of metagenomic profiles (STAMP, v2.1.3) to perform Welch’s t-test and study taxonomic differences among groups. Statistical significance was defined as a *p*-value less than 0.05.

## 3. Results

### 3.1. Demographic and Clinical Characteristics of Participants

Table 1 shows the demographic and clinical data of the groups of participants. The IPSS of patients with BPH (8.74 ± 6.88) and PC (6.66 ± 6.51) was significantly higher than that of the control group (2.29 ± 2.07) in both storage and voiding symptom scores. The QOLS of patients with BPH (2.56 ± 1.21) and PC (2.23 ± 1.01) reflected the discomfort they suffered compared with the control group (1.18 ± 0.44). Testosterone levels of patients with PC (307.82 ± 258.03 ng/dL) were lower than those of patients with BPH (551.67 ± 246.50 ng/dL) and the control group (565.06 ± 213.58 ng/dL). No other clinical data showed any significant differences between the groups.

### 3.2. Bioinformatics Analysis

**Figure 1:**
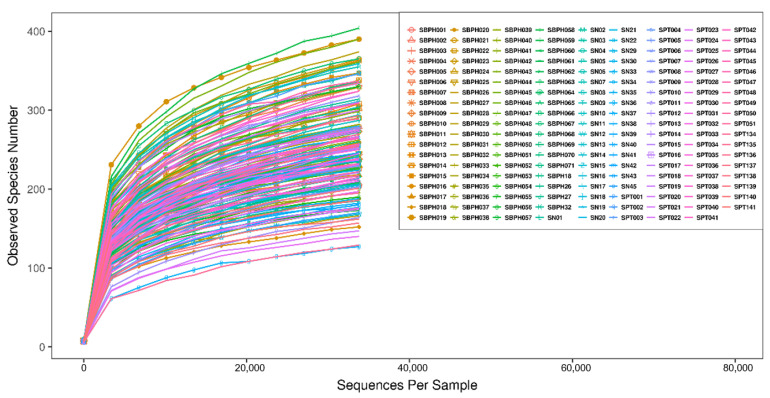
Rarefaction curve of stool samples. Sequence number threshold of the stool sample was set at 33,800. Each line represents a stool sample collected from a project participant. SBPH: Stool from a patient with BPH. SN: Stool from control group. SPT: Stool from a patient with prostate cancer.

**Figure 2:**
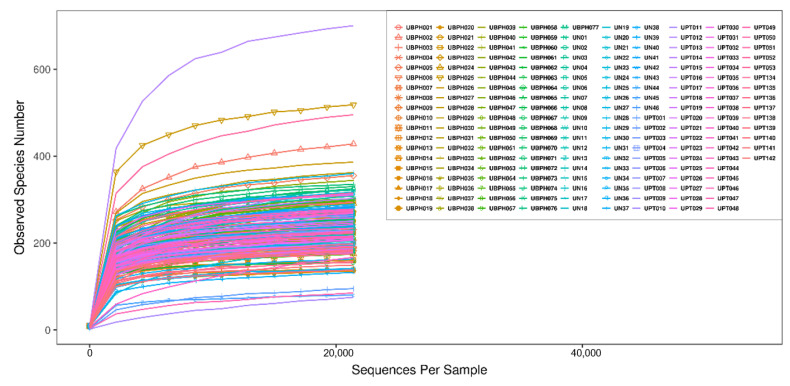
Rarefaction curve of urine samples. Sequence number threshold of the urine sample was set at 21,410. Each line represents a urine sample collected from a project participant. UBPH: Urine from a patient with BPH. UN: Urine from control group. UPT: Urine from a patient with prostate cancer.

**Figure 3:**
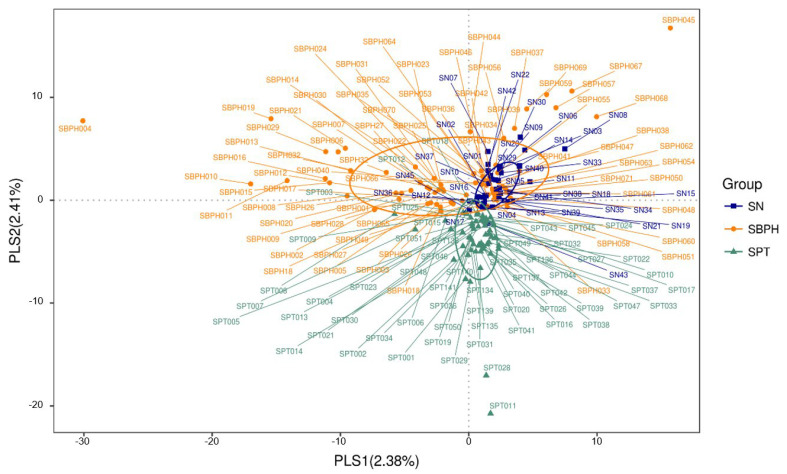
Stool sample PLS-DA plot for visualizing the difference among three groups. Each spot represents a stool sample collected from a project participant. SBPH: Stool from a patient with BPH. SN: Stool from control group. SPT: Stool from a patient with prostate cancer.

**Figure 4:**
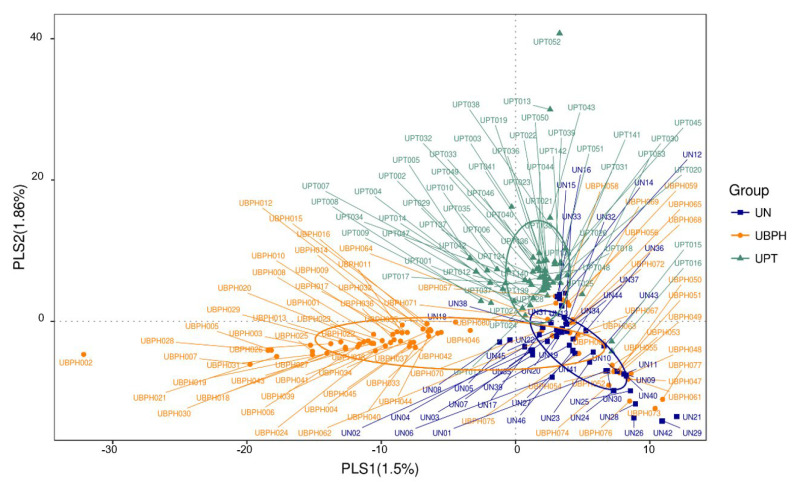
Urine sample PLS-DA plot for visualizing the difference among three groups. Each spot represents a urine sample collected from a project participant. UBPH: Urine from a patient with BPH. UN: Urine from control group. UPT: Urine from a patient with prostate cancer.

The rarefaction curve represents the sufficiency of stool and urine sample sizes as it flattens (Figure 1 and Figure 2). The sequence number threshold of the urine and stool samples were set at 21,410 and 33,800, respectively. According to the ANOSIM analysis (Table 2), we did not find a significant difference among stool samples. That is, the digestive tract microbiota did not differ between patients with BPH and PC and the control group. The stool sample PLS-DA plot is illustrated in Figure 3. In contrast, we noticed that the patients with BPH, patients with PC, and control groups displayed statistically significant differences in urinary tract microorganism composition. The urine sample PLS-DA plot visualized the differences among the three groups (Figure 4). Hence, we focused our study on analyzing the microbiota originating from urine samples.

**Figure 5:**
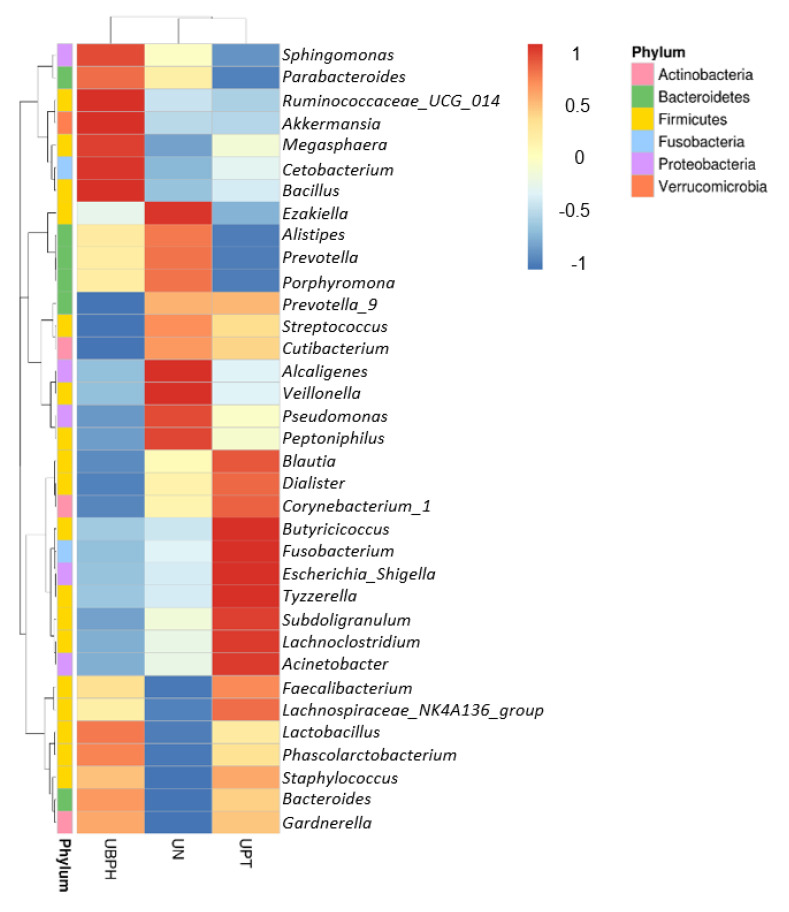
Genus heat map lists down the top 35 genera identified in urine sample. The z-score of each genus abundance in each group is shown in different color. The z-score is computed on a genus-by-genus (row-by-row) basis by subtracting the mean and then dividing by the standard deviation. The right upper -1–1 color bar stands for standardized z-score. UBPH stands for patients with BPH. UN stands for control group. UPT stands for patients with prostate cancer.

**Figure 6:**
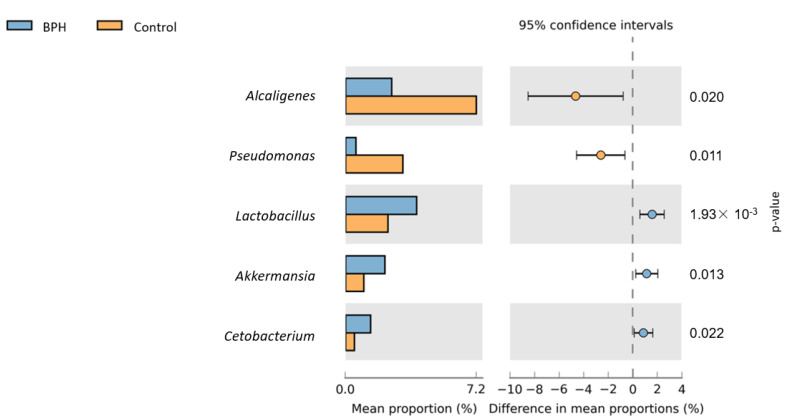
Urine sample microorganism genus relative abundance—BPH vs. Control. Left side of the figure shows mean abundance proportion. Right side shows difference in mean abundance proportions 95% confidence interval.

**Figure 7:**
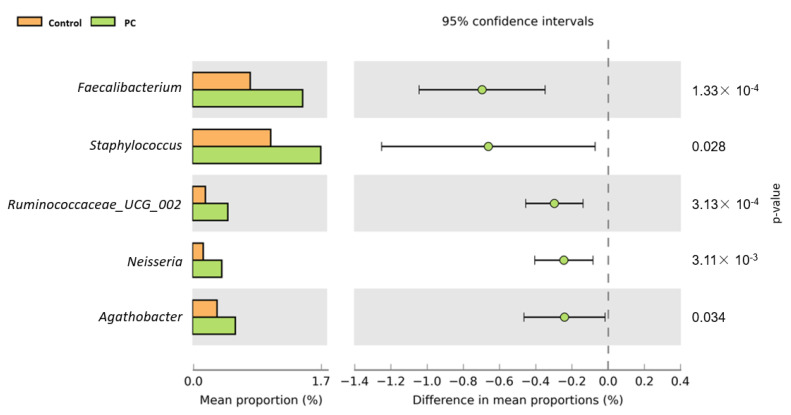
Urine sample microorganism genus relative abundance—PC vs. Control. Left side of the figure shows mean abundance proportion. Right side shows difference in mean abundance proportions 95% confidence interval.

**Figure 8:**
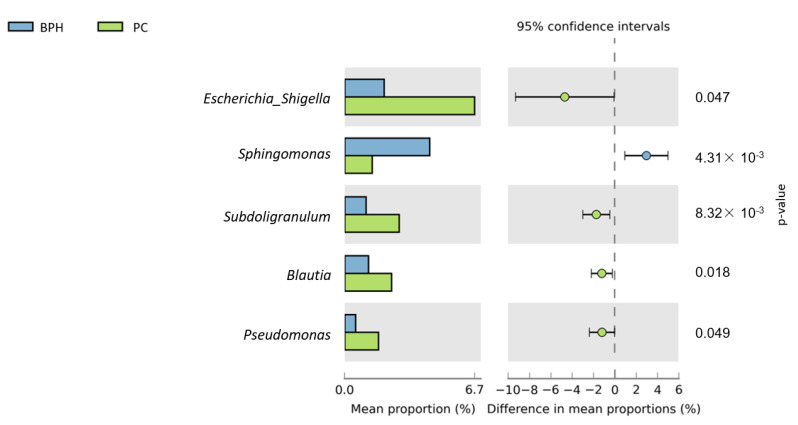
Urine sample microorganism genus relative abundance—BPH vs. PC. Left side of the figure shows mean abundance proportion. Right side shows difference in mean abundance proportions 95% confidence interval.

**Figure 9:**
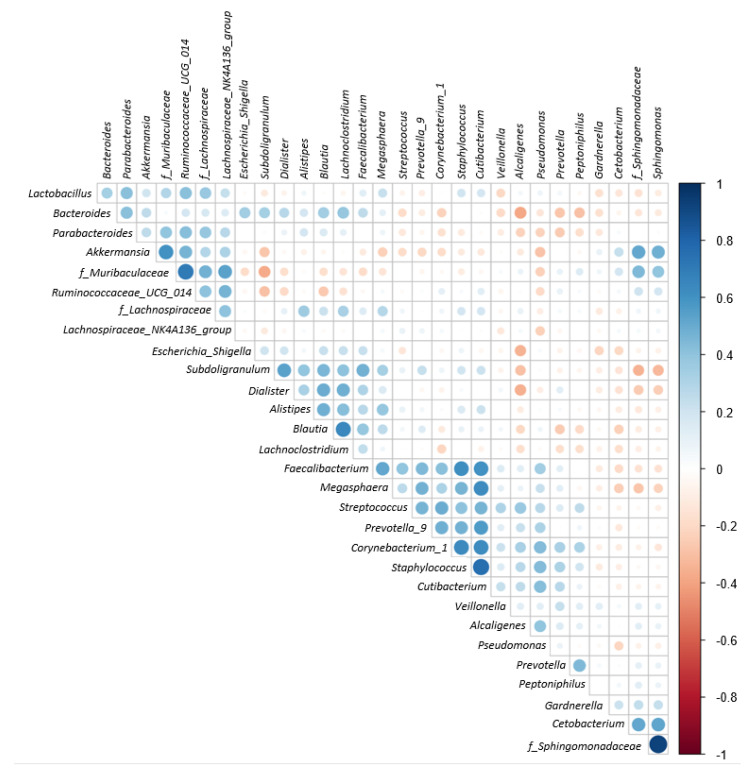
Spearman correlation coefficient plot for exploring dependency between genera. Those categories starting with “f_” means that its “family” taxonomic information is recorded instead of genus. Different color shade of right side color bar represents Spearman correlation coefficient. Blue means positive correlation; red means negative correlation.

Figure 5 shows a heat map of the top 35 genera identified in the urine sample. A standardized z-score was calculated for every genus in each of the three studied groups. We can then determine which genera thrive in the urinary tract in certain populations. In Figure 6, Welch’s t-test shows the metagenomic profiles of the microbiota genus. The top five microbial genera that showed significant differences between the patients with BPH and control groups were *Alcaligenes*, *Pseudomonas*, *Lactobacillus*, *Akkermansia*, and *Cetobacterium*. Figure 7 shows a comparison between the patients with PC and control groups. *Faecalibacterium*, *Staphylococcus*, *Ruminococcaceae_UCG_002*, *Neisseria*, and *Agathobacter* had the largest proportion differences. A comparison of the BPH and PC microorganism genera is shown in Figure 8. *Escherichia Shigella*, *Sphingomonas*, *Subdoligranulum*, *Blautia*, and *Pseudomonas* were the top five genera with differences in mean proportions. The Spearman correlation coefficient plot (Figure 9) was used to determine the dependency between genera. This information paves the way for future research on the role of individual species in the urinary tract. Our next stage of research will further correlate species abundance with clinical data, such as PSA levels and IPSS scores.

## 4. Discussion

The gut microbiota is linked to the host’s overall health because of its role in the host’s nutritional metabolism, mucosal barrier maintenance, and immunomodulation [12]. Children with aberrant gut microbiome composition during infancy are more likely to be overweight [13]. Commensal microorganisms exert their influence through their metabolites, thereby affecting the host’s metabolic function [14]. Are urinary tract diseases and symptoms related to the gut microbiota? In our study, the microorganisms identified in stool samples did not show statistically significant differences between the control group, patients with BPH, and patients with PC. However, fecal microbiota might have been an indicator of LUTS in a previous study. Holland et al. collected fecal samples from 30 patients and identified 48 fecal OTUs that correlated with LUTS, such as nocturia, storage symptoms, and voiding symptoms [15]. The composition of the gastrointestinal microbiota may be considered relevant to PC risk [16].

In contrast, the gut microbiota composition of the control group was found to be not different from that of patients with prostate disease. This may be due to the susceptibility of gut flora. Antibiotics and diet are two major factors that affect the microbiota of the digestive tract. Although patients taking antibiotics within two months of sample collection were excluded, we did not provide the same diet to all participants. This was done to maintain the practicality of this study, which may be adopted by other clinicians. Clinicians treat patients with all kinds of diets, and it is impractical to limit the patient’s diet just because physicians want to predict their prostatic disease risk by identifying their stool microbiota. Our study is one of the large pioneering studies to collect both stool and urine samples from patients with BPH and PC and to compare them using 16S rRNA sequencing. Given the adequacy of our sample size, we demonstrated that microbiota detected in stool may not be the key factor associated with the development of BPH and PC. Urine contacts the prostate gland directly, which may explain the role of the urine microbiota in prostate disease generation. Furthermore, several studies reveal the role played by the urinary tract microbiota in LUTS and prostatic disease. Bajic et al. aimed to establish an association between the lower urinary tract microbiota (LUTM) and LUTS. Although the results of voided urine sample 16S rRNA sequencing showed no statistically significant relationship between IPSS and the presence of bacteria, catheterized urine samples showed positive results. Increased IPSS in patients is related to a higher possibility of detecting LUTM in catheterized urine [17]. Besides the known pathogenic factors of BPH, such as aging and androgens [18], chronic prostatic inflammation also seems to be one of the etiologies of BPH [19]. Prostatic inflammation contributes to PC in humans as well [20]. Even though scientists and clinicians have not fully understood the association between proinflammation microbiota, BPH, and PC, genitourinary tract microbiota has been a hot research topic in prostatic disease pathophysiology in the past few years.

In our urine sample analysis, *Lactobacillus* and *Staphylococcus* were abundant genera that separately reflected statistical differences in patients with BPH and PC compared with the control group population. Yin et al. [21] reported slightly different results. The analysis of urine flora DNA sequencing showed distinct microbiota composition in patients with BPH and PC compared to the control group. The relative abundance of *Escherichia coli* was higher in the BPH and PC groups, but *Lactobacillus iners* and *Lactobacillus helveticus* thrived in healthy individuals instead of in patients with BPH and PC. Mändar et al. profiled the seminal microbiome in men with and without prostatitis. The semen of patients with chronic prostatitis was found to have contained fewer *Lactobacillus* than the semen of a healthy person [22]. Drinking fermented milk containing *Lactobacillus casei* strain Shirota has been reported to increase natural killer cell activity [23]. More specifically, an in vitro study by Horinaka et al. showed that *Lactobacillus* strains evoke tumor necrosis factor-related apoptosis-inducing ligand (TRAIL) production to strengthen natural killer cell activity against human prostate cancer PC3 cells [24].

Our study has some limitations. First, although people who received antibiotic treatment within two months before sample collection were excluded, other variables that may affect human body microbiota composition, such as constant aerobic activity [25], were not considered. Second, most patients are under different regimens for disease control, which may influence microbiota composition. A study by Li et al. presented different gut microbiome compositions in patients with PC who underwent androgen deprivation therapy and prostatectomy [26]. Furthermore, urine samples were collected from mid-term voided urine, and different collection methods, including catheterized urine collection, may identify different microbes residing in the bladder and urethra. Nevertheless, to our knowledge, this is the largest microbiota study comparing prostate diseases and a normal cohort with an adequate sample size. This may be a pioneering study for future experiments.

In conclusion, voided urine and stool samples were collected from patients with BPH, patients with PC, and a control group to study microbial composition using 16S rRNA sequencing to identify specific microbes. We did not observe statistically significant differences in stool samples, but urine samples reflected that the flora of patients with BPH and PC were distinct from those of the control group. We propose that urine microbiota may affect the development of prostate disease.

## Figures and Tables

**Table 1:** Demographic and clinical data of research participants.

	Control	BPH	PC	*p* Value
Number of Urine Sample	46	77	62	
	Mean ± SD	Mean ± SD	Mean ± SD	
Age	62.84 ± 7.70	69.44 ± 8.23	71.15 ± 7.35	<0.01
Height (m)	1.67 ± 0.06	1.67 ± 0.07	1.66 ± 0.06	0.24
Weight (kg)	69.08 ± 8.61	67.91 ± 11.67	67.36 ± 8.94	0.68
BMI	24.77 ± 2.89	24.14 ± 3.29	24.50 ± 2.68	0.51
Prostate size (gm)	N/A	42.29 ± 20.64	47.43 ± 22.35	0.19
IPSS	2.29 ± 2.07	8.74 ± 6.88	6.66 ± 6.51	<0.01
Voiding symptoms	0.44 ± 1.12	4.26 ± 4.21	2.75 ± 4.05	<0.01
Storage symptoms	1.84 ± 1.26	4.48 ± 3.11	3.90 ± 3.00	<0.01
QOLS	1.18 ± 0.44	2.56 ± 1.21	2.23 ± 1.01	<0.01
Ac sugar	109.77 ± 26.59	110.54 ± 17.22	115.48 ± 21.48	0.29
HbAlc	5.95 ± 0.53	5.81 ± 0.67	6.02 ± 0.85	0.19
Chol	185.36 ± 31.92	181.47 ± 42.32	186.68 ± 37.91	0.71
TG	104.59 ± 47.76	108.32 ± 66.26	113.80 ± 63.12	0.74
LDL	115.87 ± 28.52	110.49 ± 33.61	113.63 ± 35.21	0.68
HDL	48.77 ± 12.81	54.06 ± 24.29	50.17 ± 19.87	0.34
GOT	27.44 ± 7.69	26.00 ± 7.59	29.68 ± 18.47	0.23
GPT	31.07 ± 18.95	26.08 ± 12.78	29.90 ± 23.12	0.28
BUN	13.32 ± 3.07	15.37 ± 4.95	14.73 ± 4.21	0.08
Cr	0.96 ± 0.18	0.98 ± 0.21	1.02 ± 0.24	0.41
PSA	2.07 ± 3.21	2.99 ± 3.20	2.66 ± 8.86	0.62
Free PSA	0.60 ± 1.07	0.60 ± 0.44	0.29 ± 1.07	0.60
Testosterone (ng/dL)	565.06 ± 213.58	551.67 ± 246.50	307.82 ± 258.03	<0.01

**Table 2:** ANOSIM analysis of BPH, PC, and control group microbiota composition of urine and stool sample.

**Urine Sample**	**R-Value**	***p*-Value**
Control vs. PC	0.0491	0.0170
Control vs. BPH	0.1086	0.0010
BPH vs. PC	0.0646	0.0010
**Stool Sample**	**R-Value**	***p*-Value**
Control vs. PC	−0.0176	0.7430
Control vs. BPH	0.0239	0.2170
BPH vs. PC	0.0179	0.1030

## Data Availability

Not applicable.
